# Reciprocal Relationship between Head Size, an Autism Endophenotype, and Gene Dosage at 19p13.12 Points to *AKAP8* and *AKAP8L*

**DOI:** 10.1371/journal.pone.0129270

**Published:** 2015-06-15

**Authors:** Rebecca A. Nebel, Jill Kirschen, Jinlu Cai, Young Jae Woo, Koshi Cherian, Brett S. Abrahams

**Affiliations:** 1 Department of Genetics, Albert Einstein College of Medicine, Bronx, New York, United States of America; 2 Dominick P. Purpura Department of Neuroscience, Albert Einstein College of Medicine, Bronx, New York, United States of America; 3 Department of Medicine, Albert Einstein College of Medicine, Bronx, New York, United States of America; 4 Saul R. Korey Department of Neurology, Albert Einstein College of Medicine and Montefiore Medical Center, Bronx, New York, United States of America; 5 Epilepsy Management Center, Albert Einstein College of Medicine and Montefiore Medical Center, Bronx, New York, United States of America; 6 Department of Pediatrics, Albert Einstein College of Medicine and Montefiore Medical Center, Bronx, New York, United States of America; Osaka University, JAPAN

## Abstract

Microcephaly and macrocephaly are overrepresented in individuals with autism and are thought to be disease-related risk factors or endophenotypes. Analysis of DNA microarray results from a family with a low functioning autistic child determined that the proband and two additional unaffected family members who carry a rare inherited 760 kb duplication of unknown clinical significance at 19p13.12 are macrocephalic. Consideration alongside overlapping deletion and duplication events in the literature provides support for a strong relationship between gene dosage at this locus and head size, with losses and gains associated with microcephaly (p=1.11x10^-11^) and macrocephaly (p=2.47x10^-11^), respectively. Data support A kinase anchor protein 8 and 8-like (*AKAP8* and *AKAP8L*) as candidate genes involved in regulation of head growth, an interesting finding given previous work implicating the AKAP gene family in autism. Towards determination of which of *AKAP8* and *AKAP8L* may be involved in the modulation of head size and risk for disease, we analyzed exome sequencing data for 693 autism families (2591 individuals) where head circumference data were available. No predicted loss of function variants were observed, precluding insights into relationship to head size, but highlighting strong evolutionary conservation. Taken together, findings support the idea that gene dosage at 19p13.12, and *AKAP8* and/or *AKAP8L* in particular, play an important role in modulation of head size and may contribute to autism risk. Exome sequencing of the family also identified a rare inherited variant predicted to disrupt splicing of *TPTE* / *PTEN2*, a *PTEN* homologue, which may likewise contribute to both macrocephaly and autism risk.

## Introduction

The autism spectrum disorders are defined by impairments in social communication and restricted or stereotypical interests or behaviors. According to the Center for Disease Control and Prevention, these disorders are common, with a reported prevalence of 1/68 [1]. Genetic analyses have identified a large and growing number of genes and loci that are known to be risk associated [2,3], however, it is now well recognized that the relationship between genetic variation and clinical diagnosis is inexact. In fact, many variants first identified in autism have been observed subsequently in individuals with intellectual disability (ID) and schizophrenia [4].

Many have sought to leverage this apparent blurring of disease boundaries by seeking out loci that map better onto disease-related traits. This has been met with success in studies of social cognition [5,6] and language performance [7–9]. Another such autism-related trait is head size. Larger than average heads were observed by Leo Kanner in his initial description of children with autism [10], and subsequent work has found additional support for involvement [11–13]. Reported rates of macrocephaly in the autism population are 14–24% [11,14–16], while reported rates of microcephaly vary from 3–15% [11,14,15]. Although the link between extreme head size and autism is clearly established, how macrocephaly and microcephaly may contribute to autism risk remains unclear. Some believe that people with autism and macrocephaly or autism and microcephaly represent different autism subgroups [11,14,17]. Furthermore, it is hypothesized that macrocephaly and microcephaly may arise from defects in neural progenitor proliferation and/or synaptic pruning, which in turn can affect brain growth and brain structure [18,19].

Many genes have been shown to be associated with extreme head size and autism with one of the most well established genes being *PTEN*. Mutations in *PTEN* have been associated with autism and macrocephaly [20], and it is currently estimated that ~5% of individuals with autism have a mutation in *PTEN* [21]. Rare mutations in other autism related genes such as *CNTNAP2* and *CHD8* are also associated with both autism and macrocephaly [22,23],while mutations in the risk genes *AUTS2* and *DHCR7* give rise to autism and microcephaly [24–26]. Another established neurodevelopmental disease risk loci, 16p11.2, causes a mirror effect with regards to gene dosage and head size. Patients with a 16p11.2 deletion often have macrocephaly while duplication patients often have microcephaly [27]. A similar phenomenon was seen within the 1q21.1 risk locus, whereby deletions and duplications give rise to microcephaly and macrocephaly, respectively [28]. Furthermore, a relationship between dosage and head size is observed in individuals with autism associated structural variants at 17q12 and 22q13.3 (Phelan-McDermid Syndrome), where deletions at both loci are associated with macrocephaly [29,30].

We present here a patient with autism and ID with an inherited duplication at 19p13.12 and discuss the relationship between altered gene dosage at this locus, involving *AKAP8* and/or *AKAP8L* in particular, and each of head size and autism. Additionally, we identified a rare maternally inherited variant in *TPTE / PTEN2*, a *PTEN* homologue, via exome sequencing of the proband and his family and propose that disruption of this gene may also contribute to regulation of head size and perhaps autism risk.

## Materials and Methods

### Ethics Statement

The Tg64 family was enrolled with written informed consent through the Department of Genetics at Albert Einstein College of Medicine, Bronx, NY (Internal Review Board approved program, protocol #20110456). For minors enrolled in this study, informed written consent was obtained via a parent. All consent forms are stored in a locked filing cabinet and uploaded online to a HIPPA compliant password protected database. This consent procedure was executed as written in our Internal Review Board approved program. The individuals in this manuscript have given written informed consent (as outlined in PLOS consent form) to publish these case details.

### Array CGH and FISH Confirmation

Genomic DNA was isolated from blood using the Puregene Genomic DNA Purification kit (Gentra, Minneapolis, MN) and aCGH performed in the context of a clinical evaluation using a custom 44k Agilent array (Santa Clara, CA). Probes were placed at 5–10 kb intervals in subtelomeric, pericentromeric, and known microdeletion/microduplication regions, providing ~50 kb resolution. Additional probes were placed every 50–100 kb across the remaining euchromatic genome resulting in a ~500 kb resolution. Samples were labeled and hybridized, washed, and scanned (CGH Analytic 3.5.14 software) according to the manufacturer’s protocol. FISH confirmation of the duplication was done by Quest Diagnostics (Madison, NJ) using the DNA probe RP11-637P24.

### Additional Clinical Genetic Testing

Standard karyotyping (550–650 band resolution) was carried out by G banding by Quest Diagnostics on fixed cells. A subtelomeric FISH assay was also performed by Quest Diagnostics with ToTelVysion sub-telomeric probes (Abbot Molecular, Abbot Park, IL). Fragile X testing was performed by Quest Diagnostics via Southern blot hybridization. MELAS (mitochondrial encephalomyopathy, lactic acidosis, and stroke-like episodes) and NARP (neuropathy, ataxia, and retinitis pigmentosa) testing were performed at Columbia University (NY, NY) by PCR and RFLP analysis of the mitochondrial genes *MTTL1* (NC_012920.1:m.3243A>G, dbSNP:rs199474657) and *MTATP6* (NC_012920.1:m.[8993T>G; 8993T>C], dbSNP:rs199476133) for MELAS and NARP, respectively. JNCL (juvenile neuronal ceroid lipofuscinosis) testing was performed at IBR-Specialty Clinical Laboratories (Staten Island, NY) via PCR testing for a 1.02 kb deletion within the *CLN3* gene (NC_000016.10:g.28485965_28486930del966).

### Affy6.0 Arrays

Genomic DNA was isolated from blood using the Puregene Genomic DNA Purification kit as described above. Affymetrix 6.0 microarrays (946 000 CNV and 906 600 SNP probes) were processed according to manufacturer protocols (www.affymetrix.com). Arrays were washed on an Affymetrix fluidics station and scanned using a GeneChip Scanner 3000 7G; image intensities were extracted as. CEL files. Quality control, genotype calling, and copy number analysis were done using the Affymetrix Genotyping Console (v4.1.4). The Birdseed algorithm (v2) was used for genotype calling and the CN5 algorithm was used for copy number analysis. Data was visualized using Affymetrix’s Chromosome Analysis Suite (ChAS) Software (v2.1).

### Exome Sequencing

Genomic DNA was isolated from blood using the Puregene Genomic DNA Purification kit as described above. Libraries were then generated according to the manufacturer’s instructions using the TruSeq DNA Sample Preparation kit (Illumina, San Diego, CA). Capture was performed using the NimbleGen SeqCap EZ Human Exome v2.0 kit (Roche, Basel, Switzerland), which targets 34 Mb corresponding to ~180 000 coding exons / 20 000 genes. Paired-end sequencing was performed using an Illumina HiSeq2000 instrument to generate 76 base pair reads. Sequence analysis was performed in the Albert Einstein Computational Core Facility. Briefly, raw sequence data were aligned to the human reference genome (Hg19) using BWA (v0.5.9) [31,32], and PCR duplicates were eliminated with Picard MarkDuplicates (v1.45) (http://broadinstitute.github.io/picard/). Local re-alignment and base quality recalibration was then carried out using GATK (v1.5) [33,34]. BAM files were merged by Picard and GATK was used for SNP/INDEL detection. This was followed by the determination of minor allele frequencies via dB SNP (v131) and 1000 Genomes Project (v2010.12 release) and prediction of the functional impact of nonsynonymous variants by BLOSUM62, Polyphen2, and SIFT [35–38]. Variants with a read depth ≥10 were considered for review. Results from a meta-analysis of separate exome sequencing data for 2591 individuals from the Simons Simplex Collection [39] (693 carefully phenotyped families with a single autistic child in each) were also reviewed for predicted loss of function variants within *AKAP8 and AKAP8L*. For this dataset, only variants with a read depth ≥20 were considered for review.

### Validation of Exome Sequencing Variants

Validation of variants identified in exome sequencing was done by performing Sanger sequencing using an ABI 3730 on PCR products generated using the following primers:

*ARGHAP11A* (chr15:32920998; hg19):

F- TGTGAAGTTGTAATTGCTTATGCC

R- TTGAACTATTTTCACACGCTTA

*ARHGAP11A* (chr15:32927988; hg19):

F- TCCCTTTTTAAATCAGCTAAAGATT

R- TCTGGGCTAAAAAGCAAACC

*TPTE / PTEN2* (chr21:10910401–10910402; hg19):

F-TTTTTTAGCATCTTGACTTTGTG

R-GTCTCAGAAAACAAAAAGCAAATGT

### Variant Submission

Copy number and sequence level variants identified and confirmed in the Tg64 family have been submitted to ClinVar (http://www.ncbi.nlm.nih.gov/clinvar/); Accession numbers SCV000212157, SCV000212158, and SCV000212159 correspond to variants in *ARHGAP11A* (NP_055598.1:p.[Arg311Ile];[Ser452Cys], *TPTE / PTEN2* (NM_199261.3:c.1357-3_1357-2delTA), and the 19p13.12 duplication, respectively.

### Normalization of Head Circumference

Published reference data were used to convert head circumference (measured in centimeters) into standardized scores. Age and gender based distributions were used for individuals under 18 years of age [40] and height and gender based norms used for individuals over 18 years of age [41].

### Subject Photographs

A 3dMDface System (Atlanta, Georgia) was used to capture subject photographs as described elsewhere [42,43]. Images were viewed in the 3dMDVultusViewer.

### Literature and DECIPHER Database Search

Additional overlapping cases were found in the literature using the search term “19p13.12”. Overlapping cases in DECIPHER were found using the search term “chr19:15051982–15809751”. Only cases where head circumference data were available or mention of clinical evaluation of head size were included in our analyses.

### Statistical Analysis

To determine the likelihood of seeing frequencies of microcephaly and macrocephaly in individuals with structural variants at 19p13.12 by chance alone, we compared observed counts to the expected (one-tailed) binomial distributions. Calculations were based on the assumption that head circumference is normally distributed within a population.

## Results

### Clinical Report

A 3.5-year-old male of African and European descent (Tg64.001), the second child born in an unremarkable pregnancy to healthy non-consanguineous parents, was seen at the Children’s Hospital at Montefiore. He presented with autism, developmental delay, and macrocephaly. Supraventricular tachycardia was observed at birth but later resolved. At age 11, the proband was additionally given a diagnosis of ID. At 15 years old, an MRI revealed maldevelopment of the posterior cerebral white matter and a CT head scan revealed optic atrophy.

### Molecular Findings

**Fig 1:**
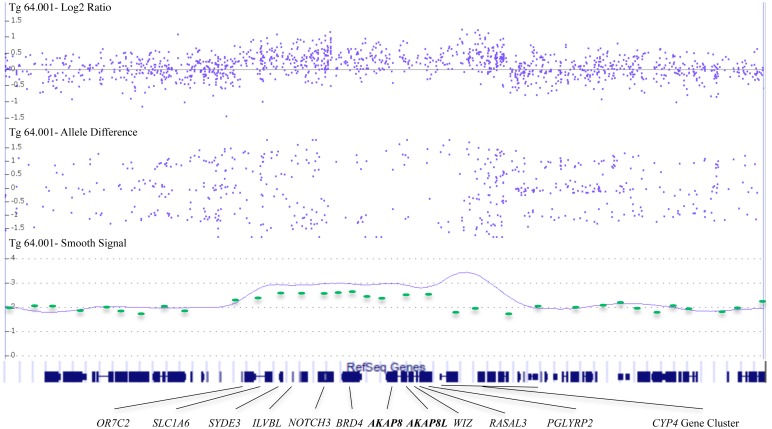
Chr19p13.12 duplication breakpoints. Results from an Affy 6.0 SNP array of Tg64.001 reveal a 760 kb duplication at 19p13.12. The top panel displays the log2 ratio of normalized intensity. The middle panel shows the difference of A allele signal and B allele signal. The bottom panel displays a Gaussian smoothed copy number estimate. Intensity values for probes included on the 44k Agilent array used in initial clinical evaluation are overlaid in green ovals.

**Fig 2:**
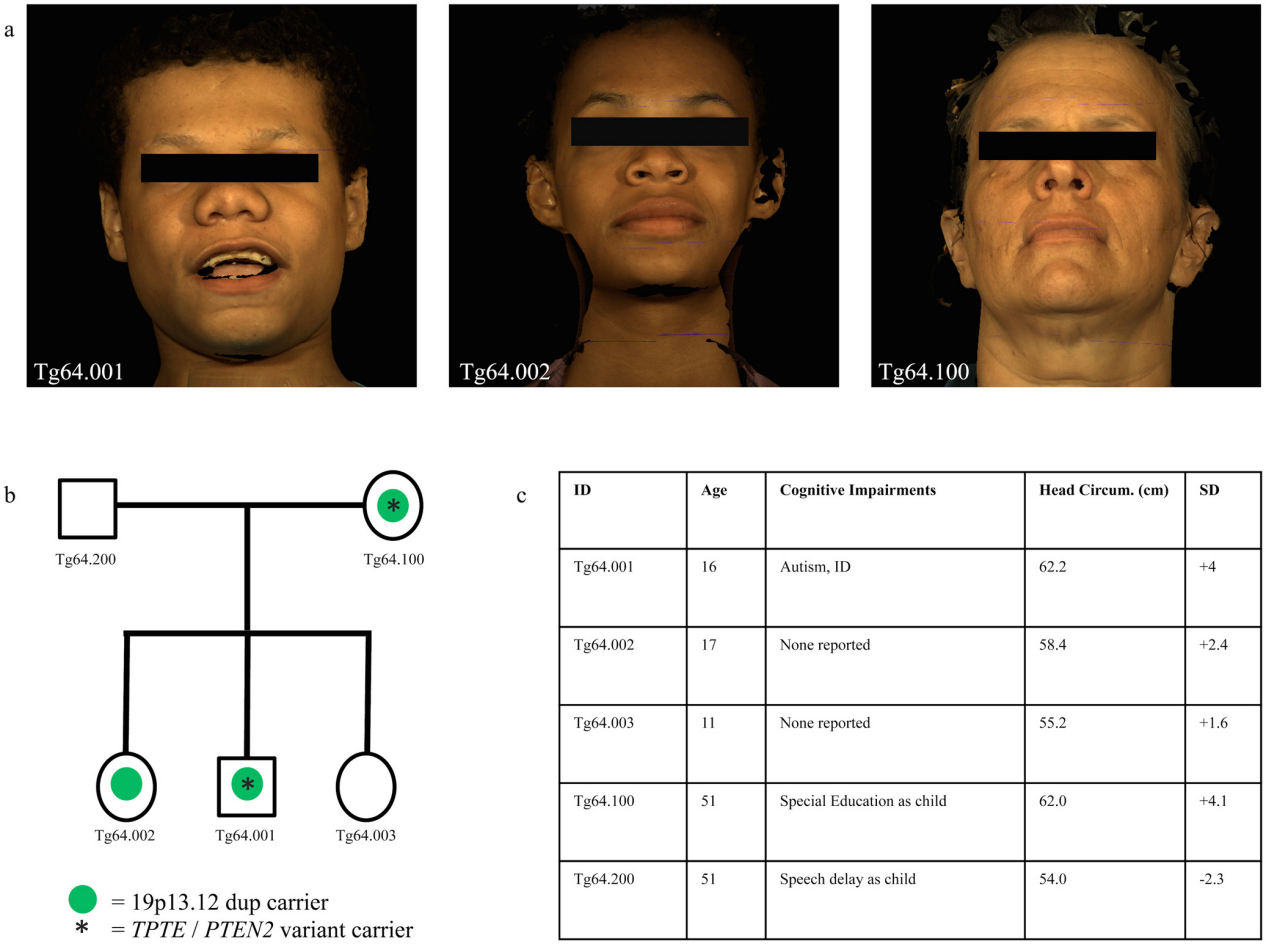
Characterization the of Tg64 family. (a) 3dMD photos of all Tg64 duplication carriers: proband (left), carrier sister (middle), carrier mother (right). (b) Carrier status of Tg64 family members for the gain observed at 19p13.12 (chr19:15051982–15809751; hg19) and sequence variant at *TPTE / PTEN2* (NM_199261.3:c.1357-3_1357-2delTA). (c) Summary of head circumference measurements and developmental concerns for Tg64 family members.

Standard karyotyping (550 band resolution), subtelomeric FISH, and testing for Fragile X, MELAS, NARP, and JNCL syndromes revealed no abnormalities in the proband. A custom 44k Agilent array, however, identified a 15 gene 645 kb duplication of unknown clinical significance at 19p13.12 (Fig 1). The duplication was confirmed by FISH and familial testing identified the identical duplication in the proband’s mother (Tg64.100), and in one of his two sisters (Tg64.002). Breakpoints were subsequently refined using an Affy 6.0 SNP array (Fig 1) to a 19 gene 760 kb region (chr19:15051982–15809751; hg19). Reexamination of available clinical data revealed macrocephaly not only in the carrier proband, but also in the two additional familial carriers, consistent with a relationship between macrocephaly and duplication status (Fig 2). Although the proband’s carrier mother was enrolled in special education classes during her schooling, his carrier sister (now in college) did not encounter such academic difficulties.

Beyond the carriers described here, examination of the literature [44–50] and the DECIPHER database [51] identified an additional 16 individuals harboring overlapping copy number variants. As summarized in Table 1, review of clinical data for all available cases suggests a gene dosage effect whereby 8 of 11 individuals with losses are microcephalic (<2 SD) (p = 1.11x10^-11^) and 7 of 8 individuals harboring gains are macrocephalic (>2 SD) (p = 2.47x10^-11^). Two non-microcephalic deletion carriers are in the 10^th^ centile or lower for head size [46,49]. No relationship to carrier status, however, was observed for either DECIPHER ID 257523 (duplication) or 265764 (deletion) where microcephaly and normal head size were observed, respectively.

**Table 1:** Summary of cytogenetic and clinical findings in cases with overlapping events at 19p13.12.

Case ID	Event Type	Coordinates (Hg19)	Size (Mb)	Inheritance	Gender	Head Size	Developmental Status
DECIPHER 257523	Gain	12.84–15.93	3.09	*de novo*	M	Micro	Intellectual Disability
Gallant et al., 2011	Loss	13.90–16.52	2.62	*de novo*	F	Micro	Unavailable
DECIPHER 285763	Loss	13.93–16.32	2.39	*de novo*	F	Micro	Intellectual Disability
DECIPHER 283124	Loss	13.93–16.32	2.39	*de novo*	F	Micro	Intellectual Disability
DECIPHER 284366	Gain	13.99–24.30	10.31	Unknown	F	Macro	Global Developmental Delay
Engels et al., 2007/Bonaglia et al. 2010	Loss	14.10–16.67	2.57	Unknown	F	Micro	Intellectual Disability
Bonaglia et al., 2010	Loss	14.27–16.19	1.92	Mat. Inherited	M	5–10 centile	Intellectual Disability
Van der Aa et al., 2010/DECIPHER 255743	Loss	14.38–15.49	1.11	*de novo*	M	Micro at birth	Intellectual Disability
DECIPHER 249355	Loss	14.66–15.66	1.00	Unknown	F	Micro	Unavailable
DECIPHER 249428	Gain	15.05–16.03	0.98	Inherited-parental origin unknown	M	Macro	Intellectual Disability
Sanders et al., 2011	Gain	15.05–15.89	0.84	*de novo*	M	Macro	Unavailable
Tg64.100 (Carrier Mother)	Gain	15.05–15.81	0.76	Unknown	F	Macro	Special Education as Child
Tg64.002 (Carrier Sib)	Gain	15.05–15.81	0.76	Mat. Inherited	F	Macro	Typically Developing
Tg64.001 (index)	Gain	15.05–15.81	0.76	Mat. Inherited	M	Macro	Intellectual Disability
DECIPHER 265764	Loss	15.05–16.24	1.19	*de novo*	M	Normal	Intellectual Disability
Jelsig et al., 2012	Loss	15.18–16.62	1.44	*de novo*	M	Micro	Intellectual Disability
DECIPHER 250827	Gain	15.18–16.46	1.28	*de novo*	F	Macro	Intellectual Disability
DECIPHER 255839	Loss	15.19–16.62	1.43	*de novo*	M	Micro	Intellectual Disability
Kosaki et al., 2011	Loss	15.44–16.20	0.76	*de novo*	F	10th centile	Mild Developmental Delay

**Fig 3:**
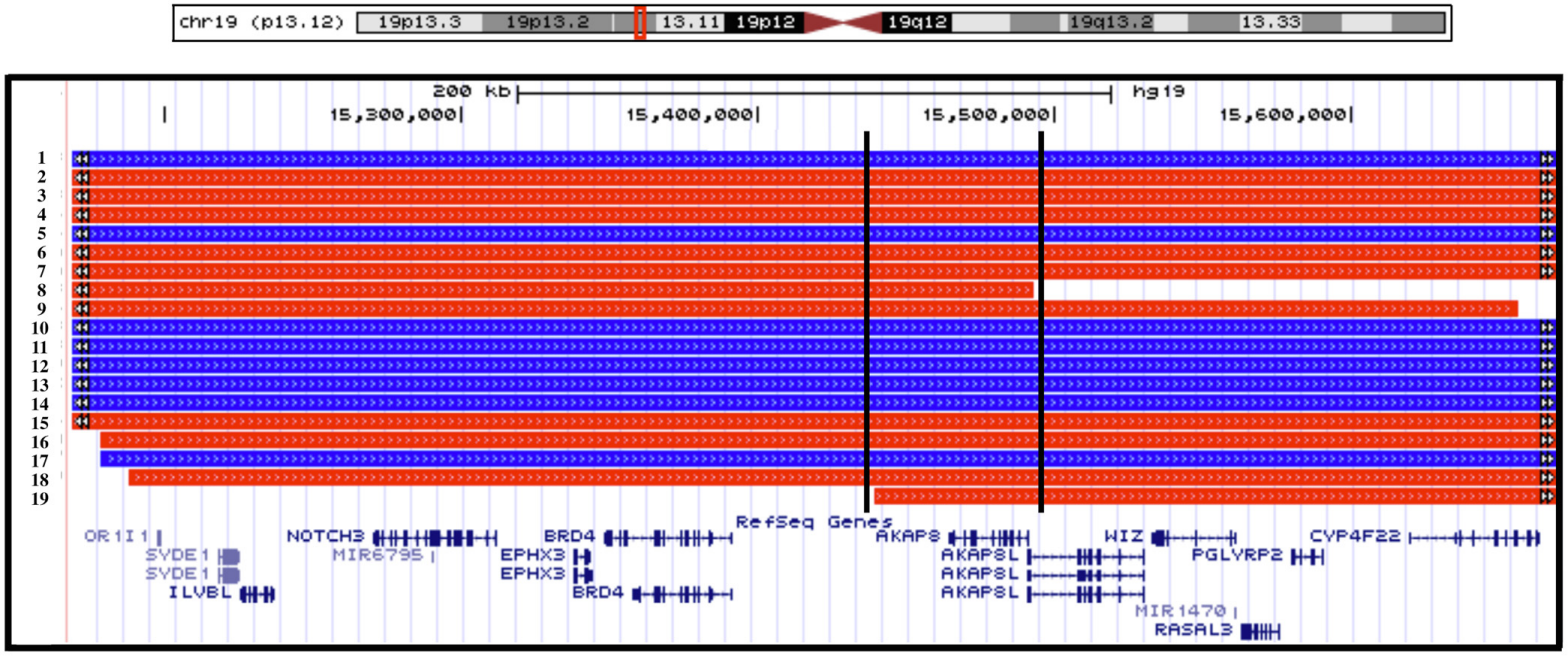
Summary of cases with copy number variants at 19p13.12 in relation to RefSeq genes. Black bars represent the critical interval breakpoints. Deletions and duplications are presented in red and blue, respectively. Cases are encoded as follows: 1- DECIPHER 257523; 2- Gallant et al., 2011; 3- DECIPHER 285763; 4- DECIPHER 283124; 5- DECIPHER 284366; 6- Engels et al., 2007/Bonaglia et al., 2010; 7- Bonaglia et al., 2010; 8- Van der Aa et al., 2010/DECIPHER 255743; 9- DECIPHER 249355; 10- DECIPHER 249428; 11- Sanders et al., 2011; 12- Tg64.100; 13- Tg64.002; 14- Tg64.001; 15- DECIPHER 265764; 16- Jelsig et al., 2012; 17- DECIPHER 250827; 18- DECIPHER 255839; 19- Kosaki et al., 2011.

Comparison of breakpoints in carrier individuals point to a 54 kb critical region (chr19:15439339–15492848; hg19) consisting of two genes: *AKAP8* and *AKAP8L* (Fig 3). Although the number of informative cases is small, this relationship of gene dosage and head size was observed in individuals of different genders and ethnicities. Almost all cases with deletions or duplications that span the locus are reported to have some degree of developmental delay (15/16 informative cases), with many having ID (12/16 informative cases), although neither was observed in Tg64.002.

In an attempt to explore the possible role of *AKAP8* and *AKAP8L* in the modulation of head size, we looked for stop gains and splice site mutations in exome sequencing data from 2591 individuals where head size was available. These analyses were non informative, as no events likely to be deleterious were observed in either gene. The absence of any disruptive variants in this large number of individuals is, however, consistent with strong evolutionary pressure on both genes. Additionally, interrogation of the Database of Genomic Variants (http://dgv.tcag.ca/dgv/app/homev.20141016release) [52] failed to identify any exonic duplications overlapping *AKAP8 or 8L*, but did show three deletions, a 1900 bp event across *AKAP8*, a 183 kb event spanning *AKAP8 and AKAP8L*, and a 1.8 Mb even spanning *AKAP8*, *AKAP8L*, and 4 other genes in the region. Although head size and other phenotypic information for individuals in which these events were identified is unknown, these data show that structural variants at the locus are rare in the general population. Lastly, GWASs for brain and intracranial volume on data from more than 15,000 people failed to see any common variant signal within this region [53,54].

Towards isolation of one or more additional variants that could better explain the clinical presentation of Tg64.001, exome sequencing was performed on the proband, mother, and father. Consideration of rare alleles (MAF < 1% based on a combination of European, African, and Asian ancestry data from the 1000 Genomes Project) predicted to be disruptive by at least two *in silico* prediction tools as well as splicing, frameshift, and non-frameshift insertion/deletion variants identified thirteen variants in twelve genes (S1 Table). Among these were two putatively deleterious variants—one inherited from each parent—in *ARHGAP11A* (NP_055598.1:p.[Arg311Ile];[Ser452Cys], dbSNP: rs372419991 and rs146176251, respectively) on 15q13.3, a gene within the RhoGAP family. Other RhoGAP family members have been implicated in autism and ID [55–58], making it an excellent candidate gene. Sanger sequencing of all family members confirmed the presence of both variants in the proband, but also found each to be present in his younger unaffected sibling (Tg64.003) ruling out any straightforward relationship to disease.

Also identified through exome sequencing was an apparent *de novo* variant in *TPTE / PTEN2* (NM_199261.3:c.1357-3_1357-2delTA). Interestingly, *TPTE / PTEN2* is a *PTEN* homologue, a gene in which mutations give rise to autism with macrocephaly [21,59,60]. Attempts at validation by Sanger sequencing determined that this rare variant in *TPTE / PTEN2* was not in fact *de novo*, but rather maternally inherited (Fig 2b). However, consistent with functionality, the observed allele is predicted to disrupt a canonical splice acceptor site and result in the elimination of 31 amino acids from a conserved C2 domain (pfam10409). Moreover, no variant at this position in *TPTE / PTEN2* was observed in the Exac database [61], a compendium of exome sequence data for more than 60,000 individuals, although the same allele we observed was identified in the 1000 Genomes Project at a frequency of 1/500 (rs149363218) (S2 Table). Consistent with a possible role for variation in *TPTE / PTEN2* in regulation of head size is the fact that the proband and his mother who each carry the gain encompassing *AKAP8 / AKAP8L* and the *TPTE / PTEN2* variant predicted to disrupt splicing have heads 1.4 SD and 1.5 SD greater than the macrocephalic sibling who carries only the gain. Exome sequencing provided no other obvious candidates to account for the proband’s clinical presentation.

## Discussion

We describe a male with autism, ID, and macrocephaly. He and two non-autistic family members have a 760 kb duplication on 19p13.12 and macrocephaly. Results presented here suggest that gene dosage at this locus, and the *AKAP8* and/or *AKAP8L* genes in particular, is strongly associated with head size. A clear positive relationship between 19p13.12 dosage and head size is observed in 17/19 informative cases. While no such relationship was seen in two carriers with overlapping copy number variants, a number of genes are thought to play a role in head size [62–64], and so it is likely there are additional variants in these individuals acting as modifiers.

AKAP8 and AKAP8L are anchoring proteins known to regulate chromatin condensation during mitosis [65,66]. AKAP8 has been shown to interact with various cyclins that regulate G1 phase and G1/S transition [67,68], and disruption of AKAP8 *in vitro* inhibits DNA replication initiation [69]. Knockdown of AKAP8 and AKAP8L together causes a G2/M arrest and aberrant chromosomal morphology [70]. Also relevant is that both *AKAP8* and *AKAP8L* are highly expressed in the human fetal brain, most prominently in transient forebrain structures. Like other microcephaly genes [64], *AKAP8* is present at high levels in the ventricular and subventricular zones, areas where the birth and division of progenitor cells occur [71]. These data suggest that altered *AKAP8* and/or *AKAP8L* dosage may impact head size through regulation of cellular proliferation in the brain. Consistent with involvement in disease, AKAP8 has been shown to interact physically with CC2D1A, an ID causing gene [72]. Moreover, a recent meta-analysis of autism GWAS data implicates the AKAP gene family in disease and suggests that many family members are targets of autism-implicated miRNAs [73].

The identification of a rare maternally inherited variant in *TPTE / PTEN* in the proband is intriguing given the well-established role for *PTEN* in autism. Previous work suggests that ~5% of children with autism carry germline mutations in *PTEN* [21], with all of these patients showing macrocephaly. *TPTE / PTEN2* expression was initially thought to be testis specific [74], however, more recent analyses shows expression in multiple structures in human fetal brain [71]. Although little is known about TPTE / PTEN2 function, *in vitro* enzymatic analyses show that this protein acts as a phosphatase to remove the 3-phosphate from substrates overlapping with those modified by PTEN [75]. Moreover, the specific variant identified here is predicted to disrupt a conserved C2 domain (pfam10409) that in PTEN is involved in the interaction with phospholipid membranes and the suppression of glioblastoma cell growth [76]. If this C2 domain acts in a similar fashion in TPTE / PTEN2, disruption may serve to accelerate cellular proliferation in brain development. Although phenotypic characterization of additional individuals who harbor rare variants in *TPTE / PTEN2* is of course required, the proband and his mother who carry both the duplication encompassing *AKAP8 / AKAP8L* and the *TPTE/ PTEN2* predicted splice variant have heads much larger (1.4 and 1.5 SD, respectively) than the macrocephalic sibling who carries only the duplication. This suggests rare variation in *TPTE / PTEN2* may be involved in biological processes underlying head growth and perhaps autism pathogenesis.

Our report, together with data from others, provides strong support for a positive relationship between gene dosage at 19p13.12 and head size where gene dosage gives rise to mirror phenotypes. Effects may be mediated by *AKAP8* and/or *AKAP8L*. Additionally, there may be a contributory role for gene dosage at this region to autism. Our results are consistent with the idea that genetic variation at 19p13.12 can play an important role in modulation of disease without being directly associated with diagnosis. Our data also suggest that *TPTE / PTEN2* may be involved in regulation of head size and perhaps autism risk.

## Supporting Information

S1 Table Exome sequencing results for Tg64 trio. Exome sequencing was performed on samples from the proband (Tg64.001), his mother (Tg64.100), and his father (Tg64.200). Variants with a read depth ≥10, a minor allele frequency <1% (based a combination of European, African, and Asian ancestry data from the 1000 Genomes Project), and with potential for functional impact are listed.
(XLSX)

S2 Table 1000 Genomes (Phase 3) allele frequencies for rs149363218 (chr21:10910401–10910402) at *TPTE / PTEN2* as a function of ethnicity. (XLSX)
